# Analytical study on couple stress flow of GO-EG and GO-W nanofluid over an extending cylinder along with variable viscosity

**DOI:** 10.1016/j.heliyon.2023.e22491

**Published:** 2023-11-21

**Authors:** Ali Rehman, Ma Chau Khun, Zabidin Salleh, Waris Khan, Maryam Sulaiman Albely, Rashid Jan, Somayah Abdualziz Alhabeeb

**Affiliations:** a Forensic Engineering Center Institute for Smart Infrastructure and Innovative Construction, Faculty of Civil Engineering, Universiti Teknologi Malaysia, Malaysia; b Department of Mathematics, Faculty of Ocean Engineering Technology and Informatics, Universiti Malaysia Terengganu, 21030, Kuala Nerus, Terengganu, Malaysia; c Department of Mathematics and Statistics, Hazara University Mansehra, 21120, Khyber Pakhtunkhwa, Pakistan; d Department of Mathematics, College of Science and Arts, Qassim University, Riyadh Al Khabra, 51961, Saudi Arabia; e Institute of Energy Infrastructure (IEI), Department of Civil Engineering, College of Engineering, Universiti Tenaga Nasional (UNITEN), Putrajaya Campus, Jalan IKRAM-UNITEN, 43000, Kajang, Selangor, Malaysia; f Department of Mathematics, College of Science and Arts, Qassim University, Al-Mithnab, 51931, Saudi Arabia

**Keywords:** GO-EG, GO-W, Couple stress parameter, Nanofluidics, Nanomaterial, Stretching cylinder, HAM BVP2.0 package

## Abstract

The main goal of this research is to present the concept of enhancing heat transfer within emerging technology. To achieve this, tiny metal and nonmetal particles ranging from 1 to 100 nm in size are introduced into base liquids. These nanoscale particles are utilized to improve the thermal performance of the liquids, leading to what are termed nanofluids. The utilization of these fluids and the examination of the flow of thin films have valuable implications across various sectors such as engineering, technology, and industries. This research focuses on analyzing the convective flow behavior of nanofluids, specifically, graphene oxide-ethylene glycol (GO−EG) and graphene oxide-water (GO−W) on a moving surface. The study investigates the impacts of magnetic fields and varying viscosity. By making use of the thermophysical characteristics of the base fluid and the nanofluid, as well as implementing a similarity transformation within the fundamental equations that govern energy and momentum, we formulate a 5th order nonlinear ordinary differential equation (NODE) to describe the velocity profile. This is combined with a second-order NODE that describes the distribution of temperature. To solve this derived NODE, we employ a method known as the Homotopy Analysis Method (HAM) for analytical solution. The impact of the relevant factors, Prandtl number, including magnetic field parameter, thickness of the liquid, couple stress parameter, temperature distribution, dynamic viscosity, and Eckert number, on the skin friction, velocity profile, and Nusselt's number are interrogated through graphical representation. The velocity field exhibits a decline as the couple stress parameter, magnetic field parameter, liquid thickness, and dynamic viscosity experience an increase. Conversely, the temperature field displays a rise as the Eckert number and dynamic viscosity experience an increase. To ensure the convergence of the issue, dual solutions of the problem are employed, and this is verified through the utilization graphs and tables. Due to the considerable challenge encountered in heat transfer applications for cooling diverse equipment and devices across industries like automotive, microelectronics, defense, and manufacturing, there is a strong expectation that this theoretical methodology could make a favorable contribution towards enhancing heat transfer efficiency. This improvement is sought to meet the requirements of the manufacturing and engineering sectors.

## 1 Introduction

Nowadays nanofluids are the most dynamic sources of energy. Nanofluids have a key role in the improvement of warmness communication strategies which have some important applications in trades and manufacturing fields. Nowadays the acquisition of energy is not a problem, the important issue is to control the feedings of energy, and this issue only be solved by implementing the upgrading heat transfer liquids to complete the mandate of the manufacturing and further associated technical turfs. The extensive study of heat transfer analysis has led researchers to tackle numerous issues. Nanofluids are a variety of fluids that consist of tiny metal atoms mixed with base liquids at concentrations of up to 5 %. They are commonly employed to improve thermal conductivity in response to the growing need for efficient cooling and heating solutions. Haq et al. [1] Conducted a study that investigated the thermal management of a partially heated trapezoidal cavity containing a water-based solution with single-wall carbon nanotubes. Rudraswamy et al. [2] organized a numerical investigation on the three-dimensional magnetohydrodynamic flow of Carreau nanoliquid on a bidirectional stretching surface. Soomro et al. [3] conducted a focused investigation on the convective heat transfer and control of nanoparticles in a Prandtl fluid model on an extending surface. On the other hand, a comprehensive study was conducted by Gul et al. [4], in which they extensively examined the consistent spreading characteristics of graphene nanoparticles. Furthermore, they explored the flow dynamics of a nanofluid containing graphene oxide-water (GO-H2O) between two surfaces in rotational motion. Yu et al. [5] organized a study to examine the thermal transport characteristics of nanofluids based on ethylene glycol, which incorporated copper nanoparticles. Xie and Chen [6] evaluated the research findings and thermal performance of nanofluids containing carbon nanotubes (CNTs). The researchers in Ref. [7] delved into the thermal conductivity of nanofluids, with a specific focus on understanding the impact of Brownian motion using fractal geometry. Similarly, Buongiorno et al. [8] conducted investigations to explore convective transport phenomena in nanofluids. On a different note, Ellahi et al. [9] examined the flow behavior of a nanofluid within a pipe, taking into consideration the influence of magnetohydrodynamics (MHD) and temperature-dependent viscosity.

Within their investigation, Khan and Pop [10] conducted a study aimed at investigating the characteristics of the boundary layer during the flow of a nanofluid over an extended surface. Correspondingly, Gul et al. [11] explored the influence of magnetohydrodynamics (MHD) on heat transfer. They accomplished this by examining a thin layer of nanoliquid that was applied as a spray onto an expanding surface, with a primary emphasis on comprehending the resulting impacts. Furthermore, Gul et al. [12] carried out research to investigate how the Hall current influences the consistent movement of a non-Newtonian nanofluid within a rotating framework. Their study accounted for the existence of both thermophoresis and Brownian motion in the system. Von Karman et al. [13] managed a research study that focused on investigating and understanding the properties and behavior of laminar and turbulent frictional flow. In an independent investigation, Rashidi et al. [14] directed their research towards the exploration of entropy production within magnetohydrodynamics (MHD) and slip flow conditions on a permeable surface that exhibited both rotation and variable properties. Similarly, Turkyilmazoglu [15], a different researcher, delved into the examination of the heat and flow transfer behaviors of nanofluids when a spinning surface was present. Sheikholeslami et al. [16] executed numerical simulations to analyze the scattering of nanofluids on an inclined spinning surface, considering its potential application in cooling systems. Shah et al. [17] engaged in a study aimed at exploring the impacts of electrical magnetohydrodynamics (MHD) and the Hall current on the flow of micropolar nanofluids between parallel plates that are in rotation. In a similar vein, Attia et al. [18] shifted their focus towards investigating the heat transfer aspects within magnetohydrodynamic (MHD) flow occurring between parallel plates. The study conducted by Vajravelua and Kumar [19] delved into the exploration of both analytical and numerical solutions for an interconnected nonlinear system that emerges within a three-dimensional rotating flow. The researchers in Ref. [20] investigated the characteristics of flow and heat transfer in nanofluids within a system undergoing rotation. They placed particular emphasis on studying the influences of a magnetic field. Mahmoodi and Kandelousi [21] employed the Differential Transform Method (DTM) to analyze how the flow and heat transfer tendencies of kerosene-alumina nanofluids manifest between two plates in rotation. Qasim et al. [22] undertook a research endeavor aimed at scrutinizing the attributes of mass and heat transfer in a nanofluid thin film situated on an elongating surface within fluctuating circumstances, utilizing Buongiorno's model. Aziz et al. [23] carried out an exploration intended to uncover the characteristics of heat and flow transfer within a thin film on an extending sheet with internal heating. Tawade and collaborators [24] delved into the examination of thin film flow and heat transfer over a stretching sheet amid varying conditions. They considered factors like internal heating, thermal radiation, and the presence of an external magnetic field. In a study conducted by Khan et al. [25], an investigation was performed concerning the flow dynamics of three non-Newtonian fluids over a surface undergoing unsteady stretching. The analysis took into consideration the attributes of thin films as well as the diverse qualities of the fluids. In a separate research effort, Qasim and Afridi [27] carried out a study to scrutinize the production of entropy and heat transfer within the boundary layer flow over a slender needle in parallel motion with a stream. Their primary focus was on the impacts of nonlinear Rosseland radiation. In a separate investigation, Afridi et al. [28] studied the non-reversible aspects of flow involving a hybrid nanofluid over a slender needle. Their analysis accounted for the impact of energy dissipation. Furthermore, Afridi et al. [29] undertook a comprehensive assessment with the aim of thoroughly understanding the production of entropy in flows with dissipation over a thin needle. They considered the alterations in thermophysical properties. Qasim et al. [30] explored the dynamics of flow around a needle moving within a dissipative fluid stream. Their examination took into consideration both the fluid's thermal conductivity and its varying viscosity. Afridi and his colleagues [31] undertook a research endeavor with the goal of investigating the entropic generation within the flow of nanofluids, suspended in carbon nanotubes (CNTs), is affected by energy dissipation and nonlinear Rosseland thermal radiation over a slender needle. In a separate study, Swamy et al. [32] focused their efforts on scrutinizing the creation of entropy and convective movement within an annular space, filled with nanoliquid consisting of alumina-water. The specific focus of their investigation was on the influence of double diffusion. In a similar vein, Pushpa et al. [33] initiated an inquiry to delve into the control of heat dissipation and buoyant flow within a porous annular chamber, employing a thin baffle for this purpose. Swamy et al. [34] conducted an analysis on the transport of energy and generation of entropy in a vertical porous annulus that was filled with a nanoliquid composed of Cu-water. In an independent research endeavor, Reddy et al. [35] investigated the convective movement generated by buoyancy within an annular space subjected to non-uniform heating. Their study encompassed various categories of hybrid nanoliquids. Additionally, Pushpa et al. [36] conducted a numerical exploration into the phenomenon of double-diffusive convection within a vertical annular enclosure. They considered the presence of a baffle within the system. Alkasasbeh et al. [43] examined the flow of a micropolar hybrid nanofluid around a solid sphere. In a similar vein, Swalmeh et al. [44] investigated the impact of inertial forces on the natural convection flow of micropolar nanofluids around a solid sphere. Continuing this line of inquiry, Swalmeh and co-authors [45] explored the impact of micro-rotation and micro-inertia on the flow of nanofluids across a heated circular cylinder oriented horizontally, within a context of free convection. Abbas et al. [46] examined the flow behavior of a power-law nanofluid with temperature-dependent properties over a varying extended surface. In a parallel study, Shatnawi et al. [47] explored hybrid nanofluid models based on the Casson formulation in the presence of induced magnetic radiative flow over a vertically permeable exponentially stretching sheet. Nazir et al. [48] conducted an analysis of the stability of a mathematical model representing a society grappling with internal extremism. Expanding on this theme, Shatnawi et al. [49] investigated the unsteady flow at a stagnation point, specifically focusing on radiative Casson hybrid nanofluids over a vertical Riga sheet. Inspired by the upstairs stated, many important uses of nanofluids in the literature, the authors are interested in the expounded fluid model. This work aims to explore the flow characteristics of GO-EG and GO-W nanofluids with variable viscosity around a translating cylinder, considering the influence of couple stress. The primary focus is to assess the heat transfer enhancement potential of these nanofluids, which hold promising applications in various technological advancements. Our research paper explores the utilization of GO-EG and GO-W nanofluids to improve heat transfer rates, offering substantial advantages to industries and engineering sectors in the foreseeable future. According to the author's knowledge from the above literature, it is observed that the GO-EG and GO-W nanofluids with variable viscosity, magnetic field around a translating cylinder, and convective boundary conditions are not yet investigated analytically. So, to fill this research gap the current research paper explains the investigation of the couple stress flow of GO-W and GO-EG nanofluid over an extending cylinder with variable viscosity. By combining the thermophysical attributes of both the nanofluid and the foundational fluid and utilizing similarity transformations within the governing equations of energy and momentum, a system of interrelated nonlinear ordinary differential equations was formulated. This system encompassed a fifth-order nonlinear ordinary differential equation that described the velocity profile, as well as a second-order nonlinear ordinary differential equation that governed the distribution of temperature. To solve these equations, the HAM (Homotopy Analysis Method), introduced by Liao et al. [26], was employed. To ensure the problem's convergence, computations for dual solutions were performed. The structure of this study is as follows: Section 2 provides an outline of the mathematical setup of the issue and the method of solution using HAM. Section 3 showcases the obtained outcomes. The analysis of these results is detailed in Section 4, while the conclusion is presented in Section 5. The nomenclature, aiding easy referencing and comprehension, is presented ahead of the references.

### 1.1 Objectives of this work

a. To increase the thermal efficiency
b. To restore the energy balance and regulate the heat transfer input.
c. The mathematical model, represented by the governing equations, is addressed using approximate analytical techniques, specifically the (HAM).
d. To communicate the idea of improving heat transfer, which finds application within emerging technological contexts.
e. Discuss the impact of various parameters obtained from temperature and velocity equations.

## 2 Formulation of the model

**Fig. 1:**
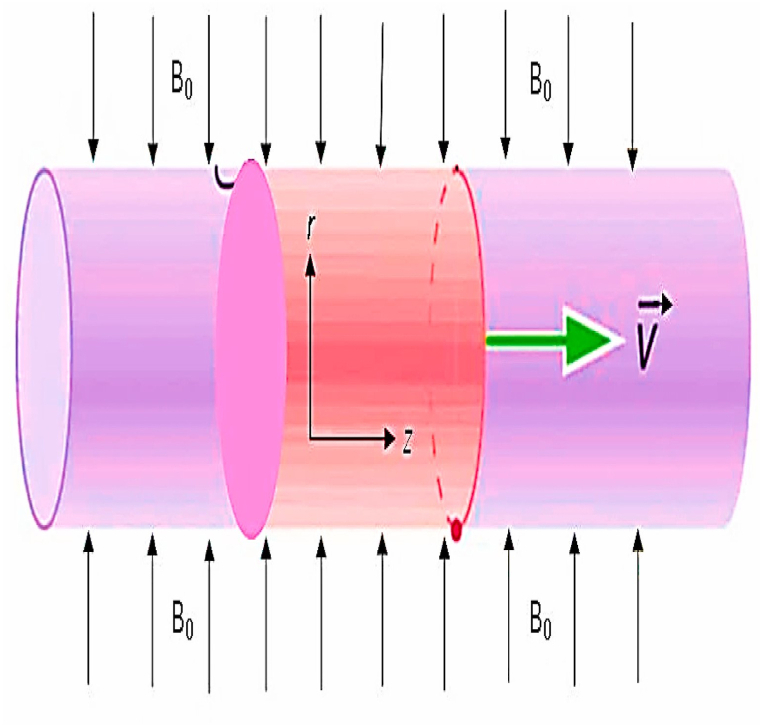
Illustration of the geometry of the model.

Take an incompressible, steady, two-dimensional flow of nanofluids, specifically graphene oxide-water (GO−W) and graphene oxide-ethylene glycol (GO−EG), over an extending cylinder. The flow's velocity field is represented by V=(u,v), and the magnetic field is represented by B=(Bx,By). The magnetic field $B_{0}$ is exerted perpendicular to the cylinder. The direction of the free stream is influenced by the movement of the surface. $T_{w}$ represents the temperature of the cylinder's wall surface and $T_{h}$ indicates the temperature of the ambient fluid with $\left(T_{w}>T_{h}\right)$. The continuity, momentum, and temperate equation. All the assumptions are taken from Gul et al. [37]. In Fig. 1, the geometry of the proposed problem has been illustrated to conceptualize the overall phenomena.

(1) $$\frac{\partial u}{\partial x}+\frac{v}{r}+\frac{\partial v}{\partial r}=0\text{,}$$

(2) $$\left(u\frac{\partial u}{\partial x}+v\frac{\partial u}{\partial r}\right)=\mu_{nf}\left(\frac{\partial^{2}u}{\partial r^{2}}+\frac{1}{r}\frac{\partial u}{\partial r}\right)+\frac{\partial \mu_{nf}}{\partial r}\frac{\partial u}{\partial r}-\frac{\sigma }{\rho_{nf}}B_{0}^{2}u-\nu \frac{\partial^{4}u}{\partial y^{4}}\text{,}$$

(3) $$\left(u\frac{\partial T}{\partial x}+v\frac{\partial T}{\partial r}\right)=\frac{k_{nf}}{{\left(\rho C_{p}\right)}_{nf}}\left(\frac{\partial^{2}T}{\partial r^{2}}+\frac{1}{r}\frac{\partial T}{\partial r}\right)+\frac{\partial k_{nf}}{\partial r}\frac{\partial T}{\partial r}+\mu_{nf}{\left(\frac{\partial u}{\partial r}\right)}^{2}\text{.}$$

The *x*-axis and *y*-axis velocities are represented as *u* and *v*, respectively. Additionally, the nanofluids density is denoted by $\rho_{nf}$ while the nanofluids viscosity is indicated by $\mu_{nf}$. In this formulation, the thermal conductivity is $k_{nf}$, $B_{0}^{2}$, is the strength of magnetic field, σ , electric condictivity and the specific heat capacity is ${\left(\rho C_{p}\right)}_{nf}$. The boundary condition for the flow problem is;

(4) $$\begin{array}{l} u=u_{w},T=T_{w},v=0,\text{at}r=R\left(x\right)\text{,}\\ \frac{\partial u}{\partial z}=\frac{\partial T}{\partial z}=0,w=u\frac{dh}{dx},\text{at}r=h \end{array}$$

The following are the provided thermal and physical properties of both the nanofluid and base fluid:

(5) $$\begin{array}{l} \rho_{nf}=\rho_{f}-\varphi \rho_{f}+\varphi \rho_{s},\mu_{nf}=\mu_{f}{\left(1-\varphi \right)}^{-2.5}\text{,}\\ k_{nf}=\frac{1-\varphi +2\left(\frac{k_{GO-EG}}{k_{GO-W}-k_{f}}\;\ln\;\frac{k_{GO-EG}+k_{f}}{2k_{f}}\right)\varphi }{1-\varphi +2\left(\frac{k_{f}}{k_{GO-EG}-k_{f}}\;\ln\;\frac{k_{GO-W}+k_{f}}{2k_{f}}\right)\varphi }k_{f}\text{,}\\ {\left(\rho C_{p}\right)}_{nf}={\left(\rho C_{p}\right)}_{f}-\varphi {\left(\rho C_{p}\right)}_{f}+\varphi {\left(\rho C_{p}\right)}_{s}\text{.} \end{array}$$

The similarity transformation is defined as

(6) $$\begin{array}{l} \psi =\nu_{f}x\;f\left(\eta \right),\eta =\frac{U_{w}r^{2}}{\nu_{f}x},\theta \left(\eta \right)=\frac{T-T_{h}}{T_{w}-T_{h}}\\ k_{nf}=k_{f}\left(1+\alpha \theta \right),\mu_{nf}=\mu_{f}\left(1+\alpha \theta \right)\text{,} \end{array}$$

ψ represent stream function which satisfy equation (1). The boundary condition of our flow problem is given in Eq (4) while the physical and thermal properties of the nanofluid and base fluid are provided in Eq (5). The velocity component $u=\frac{1}{r}\frac{\partial \psi }{\partial r},v=-\frac{1}{r}\frac{\partial \psi }{\partial x}$. To transform the basic flow equations (1)–(3) from their dimensional state to a dimensionless version, one must apply the following similarity transformations (6), as well as the thermophysical attributes of both the base fluid and nanofluid (5). Using the similarity variable η, Eq (6) ensures the continuity equation is balanced, and it alters Eqs (2), (3) into the subsequent format.

(7) $$2{\left(1-\varphi \right)}^{-2.5}\frac{1}{\left(1+\alpha \theta \right)}\left(\eta f^{\prime \prime \prime }+f^{\prime \prime }\right)+\left(1-\varphi +\varphi \frac{\rho_{s}}{\rho_{f}}\right)\left(ff^{\prime \prime }-M{f^{\prime }}^{2}\right)-Kf^{iv}=0$$

(8) $$2\left(\frac{k_{nf}}{k_{f}}\right)\left(\theta^{\prime }+\left(1+\alpha \theta \right)\eta \theta^{\prime \prime }\right)+\left(1-\varphi +\varphi \frac{{\left(\rho C_{p}\right)}_{s}}{{\left(\rho C_{p}\right)}_{f}}\right)\mathrm{Pr}\left(f\theta^{\prime }\right)+Ec{\left(1-\varphi \right)}^{2.5}\eta {\left(f^{\prime \prime }\right)}^{2}=0$$

with the following

(9) $$\begin{array}{l} f\left(c\right)=\frac{\lambda }{2}c,f^{\prime }\left(c\right)=\frac{\lambda }{2},\theta \left(c\right)=1\text{,}\\ f^{\prime \prime }\left(\beta \right)=0\text{,}f\left(\beta \right)=0\text{,}\theta^{\prime }\left(\beta \right)=0\text{.} \end{array}$$

where $\lambda =\frac{u_{w}}{U_{w}}$ are the fused velocity when λ=0 tells to a moving fluid (blassius flow) and when λ=1 tells to a motionless fluid (sakiadis flow), in our study λ≤1 is partial. The parameter β are the thickness of the liquid and is stated as

(10) $$\beta =\frac{\upsilon_{f}xh^{2}}{U_{w}},\frac{dh}{dx}=\frac{1}{2}\sqrt{\frac{\upsilon_{f}\beta }{x\;U_{w}}}\text{,}$$

The transformation develops the following dimensionless parameters are magnetic field, Prandtl number and Eckert number respectively.

(11) $$M=\frac{\sigma B_{0}^{2}}{2U_{w}\rho },\mathrm{Pr}=\frac{\mu C_{p}}{k_{f}},\text{Ec}=\frac{8U_{w}^{2}}{k_{f}\left(T_{w}-T_{h}\right)}$$

The dimensionless form of skin friction is:

(12) Re12Cf=4c12(1−φ)−2.5f″(c),

The Nusselt number's dimensionless representation is:

(13) Re−12Nu=[−2c12KnfKf]θ′(c),

where Rex=U0xvf..

## 3 Homotopy analysis method solution

To address the derived equations Eq (7) and Eq (8), in conjunction with Eq (9), we employed the approximate analytical method HAM. The solutions derived through this method are governed by a convergence-controlling auxiliary parameter h.

The initial solution is determined as provided:

(14) $$f_{0}\left(\eta \right)=-\frac{\left(\eta -\beta \right)\left(-2c^{3}+3c^{2}\beta +\eta \left(\eta -2\beta \right)\beta \right)\lambda }{4{\left(c-\beta \right)}^{3}},\theta \left(\eta \right)=1$$

where $L_{f}\text{and}L_{\theta }$, are the linear operator of velocity and temperature respectively and defined as:

(15) $$L_{f}\left(f\right)=f^{⁗}\text{and}L_{\theta }\left(\theta \right)=\theta \prime \prime \text{,}$$

such that

(16) $$L_{f}\left(c_{1}+c_{2}\eta +c_{3}\eta^{2}+c_{4}\eta^{3}\right)=0\;\text{and}L_{\theta }\left(c_{5}+c_{6}\eta \right)=0\text{,}$$

in which $c_{i}\left(i=1-6\right)$ represent the constants series solution.

Let $N_{f}$ and $N_{\theta }$ represent velocity and temperature, respectively, and both are characterized as nonlinear operators and both are defined as

(17) $$N_{f}\left[f\left(\eta ,b\right)\right]=\left(1+\alpha \theta \right)\left(\eta \frac{\partial^{3}f\left(\eta ,b\right)}{\partial \eta^{3}}+\frac{\partial^{2}f\left(\eta ,b\right)}{\partial \eta^{2}}\right)+f\left(\eta ,b\right)\frac{\partial^{2}f\left(\eta ,b\right)}{\partial \eta^{2}}-M{\left(\frac{\partial f\left(\eta ,b\right)}{\partial \eta }\right)}^{2}-K\frac{\partial^{5}f\left(\eta ,b\right)}{\partial \eta^{5}}\text{,}$$

(18) $$\begin{array}{l} N_{\theta }\left[f\left(\eta ,p\right),\theta \left(\eta ,b\right)\text{,}\right]=\left(1+\alpha \theta \right)\eta \frac{\partial^{2}\theta \left(\eta ,b\right)}{\partial \eta^{2}}+\frac{\partial \theta \left(\eta ,b\right)}{\partial \eta }+\\ \mathrm{Pr}\left(f\left(\eta ,b\right)\frac{\partial \theta \left(\eta ,b\right)}{\partial \eta }\right)+Ec\left(\eta {\left(\frac{\partial f\left(\eta ,b\right)}{\partial \eta }\right)}^{2}\right)\text{,} \end{array}$$

As per the analytical method HAM [[38], [39], [40], [41]].

The zero^th^ -order problems from Eqs. (7)–(8) are:

(19) $$\left(1-b\right)L_{f}\left\{f\left(\eta ,b\right)-f_{0}\left(\eta \right)\right\}=bh_{f}N_{f}\left\{f\left(\eta ,b\right)\right\}\text{,}$$

(20) $$\left(1-b\right)L_{\theta }\left\{\theta \left(\eta ,b\right)-\theta_{0}\left(\eta \right)\right\}=bh_{\theta }N_{\theta }\left\{f\left(\eta ,b\right),\theta \left(\eta ,b\right)\right\}\text{,}$$

The flow problem having the following boundary conditions:

(21) $${f\left(\eta ,b\right)|}_{\eta =0}=0\text{,}\;{\frac{\partial f\left(\eta ,b\right)}{\partial \eta }|}_{\eta =0}=1\text{,}{\frac{\partial^{2}f\left(\eta ,b\right)}{\partial \eta^{2}}|}_{\eta =\infty }=0\text{,}{\theta \left(\eta ,b\right)|}_{\eta =0}=1\text{,}{\begin{array}{l} \frac{\partial \theta \left(\eta ,b\right)}{\partial \eta } \end{array}|}_{\eta =\infty }=0\text{,}$$

where b∈[0,1] while $h_{f}\text{and}h_{\theta }$ are utilized to control the solution convergence. For =0andb=1 , we get:

(22) f(η,1)=f(η),andθ(η,1)=θ(η),

The Taylor's series of f(η,b)andθ(η,b) about b=0 are:

(23) $$f\left(\eta ,b\right)=f_{0}\left(\eta \right)+\sum_{m=1}^{\infty }f_{m}\left(\eta \right)b^{m}\text{,}$$

(24) $$\begin{array}{l} \theta \left(\eta ,b\right)=\theta_{0}\left(\eta \right)+\sum_{m=1}^{\infty }\theta_{m}\left(\eta \right)b^{m}\text{,} \end{array}$$

where

(25) $$f_{m}\left(\eta \right)={\frac{1}{m!}\frac{\partial f\left(\eta ,b\right)}{\partial \eta }|}_{b=0}\text{and}\theta_{m}\left(\eta \right)={\frac{1}{m!}\frac{\partial \theta \left(\eta ,b\right)}{\partial \eta }|}_{b=1}\text{,}$$

where $h_{f}$, and $h_{\theta }$ are chosen in way that (23–24) converges at b=1, and b=0 in both (23–24), we obtain

(26) $$\begin{array}{l} f\left(\eta \right)=f_{0}\left(\eta \right)+\sum_{m=2}^{\infty }f_{m}\left(\eta \right)\text{,}\\ \theta \left(\eta \right)=\theta_{0}\left(\eta \right)+\sum_{m=2}^{\infty }\theta_{m}\left(\eta \right)\text{,} \end{array}$$

The $m^{th}$-order problem for velocity and temperature hold the following:

(27) $$\begin{array}{l} L_{f}\left[f_{m}\left(\eta \right)-\varsigma_{m}f_{m-2}\left(\eta \right)\right]=h_{f}R_{m}^{f}\left(\eta \right)\text{,}\\ L_{\theta }\left[\theta_{m}\left(\eta \right)-\varsigma_{m}\theta_{m-2}\left(\eta \right)\right]=h_{\theta }R_{m}^{\theta }\left(\eta \right)\text{,} \end{array}$$

The problem having the following transform boundary condition:

(28) $$\begin{array}{l} f\left(c\right)=0\text{,}f^{\prime }\left(c\right)=0\text{,}\theta \left(c\right)=0\text{,}\\ f^{\prime \prime }\left(\beta \right)=0\text{,}f\left(\beta \right)=0\text{,}\theta^{\prime }\left(\beta \right)=0\text{.} \end{array}$$

## 4 Convergence of HAM

**Fig. 2:**
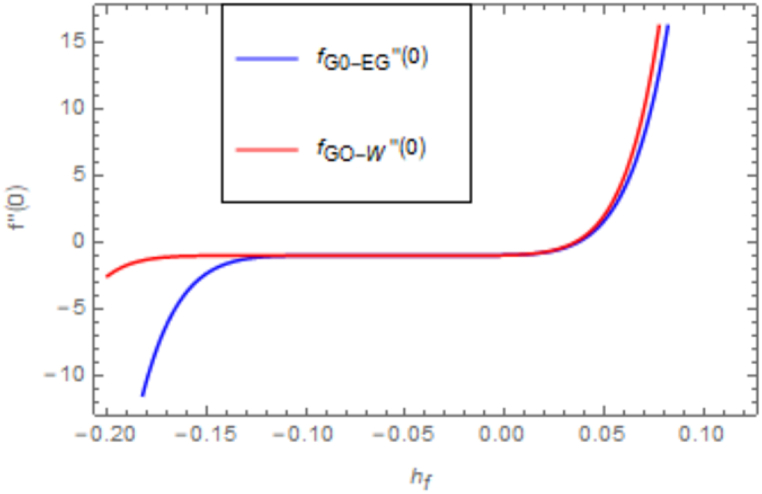
Illustration of h curve for velocity equation.

**Fig. 3:**
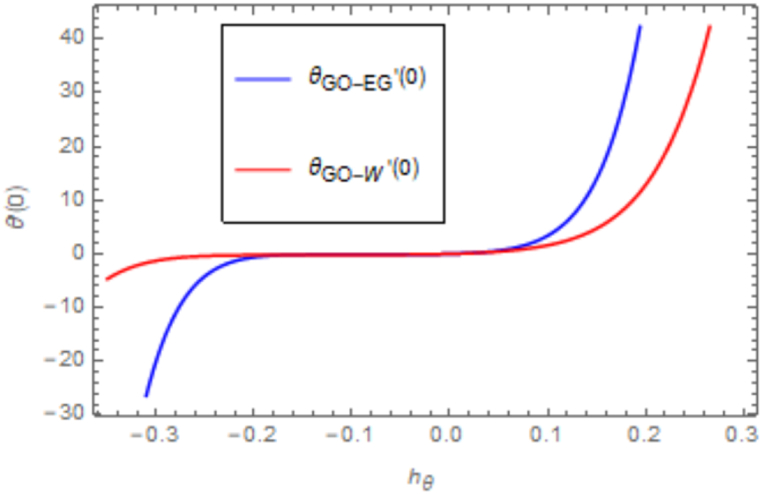
Illustration of h curve for temperature equation.

Fig. 2, Fig. 3 depict two sets of HAM-derived series solutions, one pertaining to the temperature equation and the other to the velocity equation, intended for convergence analysis. The auxiliary functions $h_{f}\text{and}h_{\theta }$, play a pivotal role in ensuring the method's convergence, serving to verify the convergence of these two solutions. Fig. 2 shows the curves we plotted for this convergence determination at the 25th order of approximations. Fig. 2 demonstrates the convergence of *f* ′′(0), converges at $-0.10\leq f^{\prime \prime }\left(0\right)\leq 0.10$ and Fig. 3 visualize the convergence of $\theta^{\prime }\left(0\right)\text{,}$ $-0.2\leq \theta^{\prime }\left(0\right)\leq 0.1$.

## 5 Results

.

## 6 Discussion

**Fig. 4:**
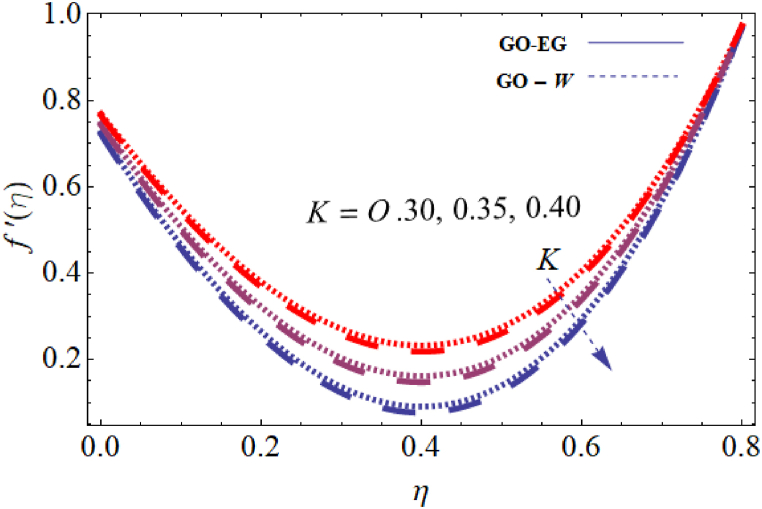
Influence of couple stress factors on the profile of velocity.

**Fig. 5:**
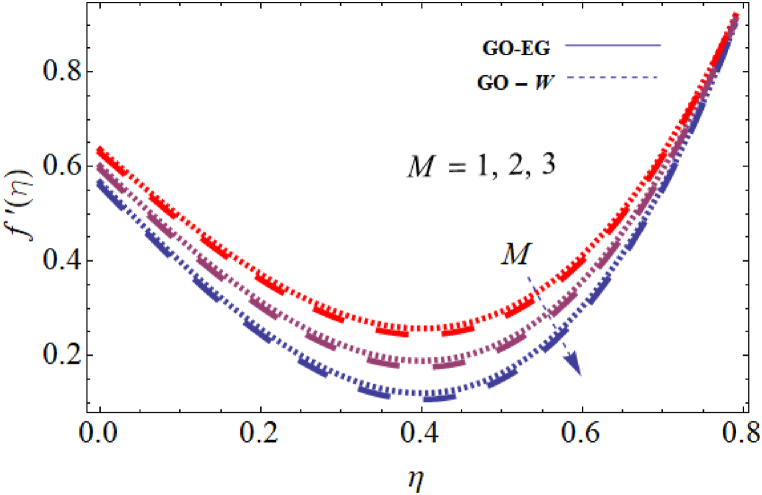
Impact of magnetic field factor on the profile of velocity.

**Fig. 6:**
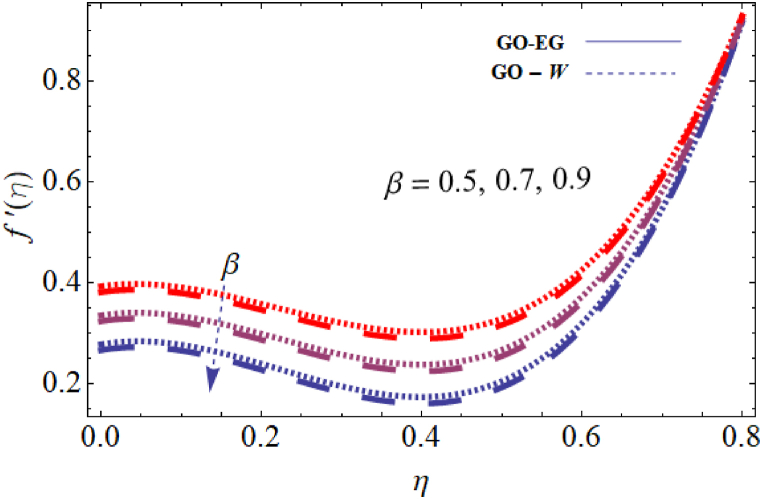
Impact of thickness of the liquid on the profile of velocity.

**Fig. 7:**
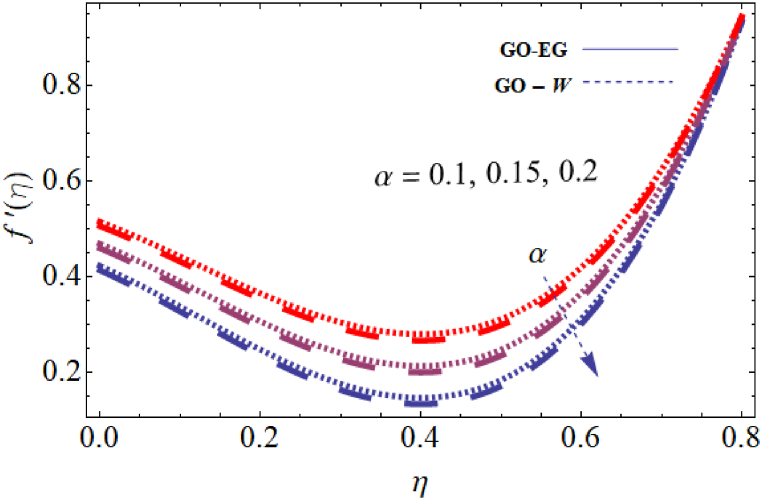
Impact of dynamic viscosity on the profile of velocity.

**Fig. 8:**
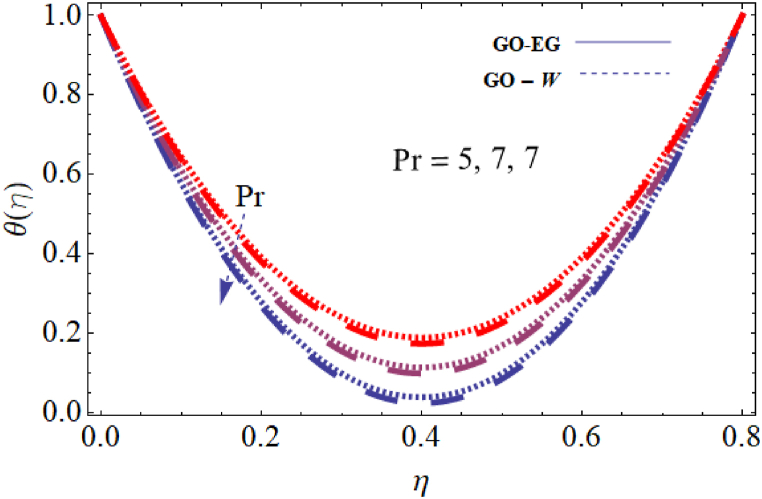
Impact of Prandtl number on temperature profile.

**Fig. 9:**
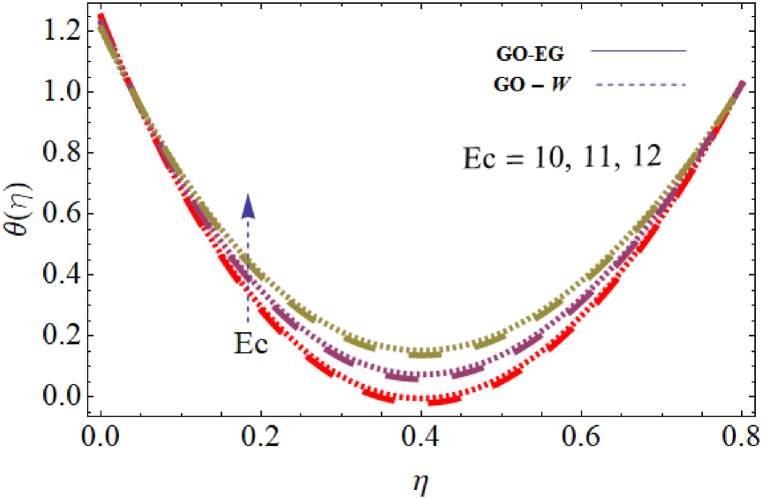
Impact of Eckert number on the profile of temperature.

**Fig. 10:**
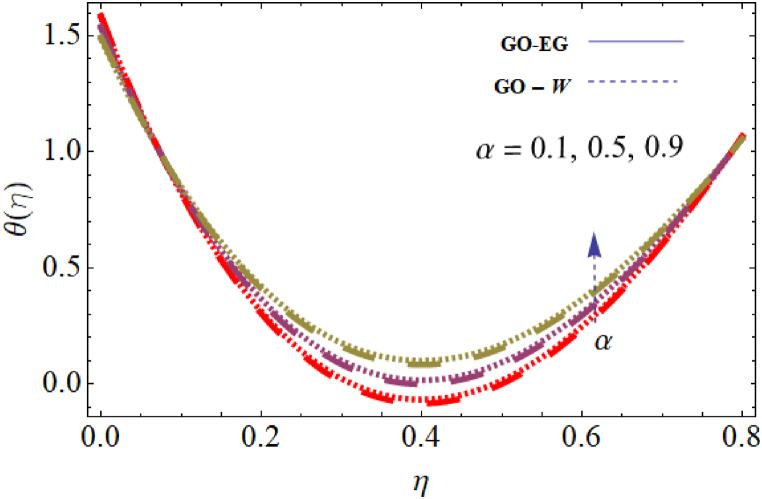
Impact of dynamic viscosity on the profile of temperature.

**Fig. 11:**
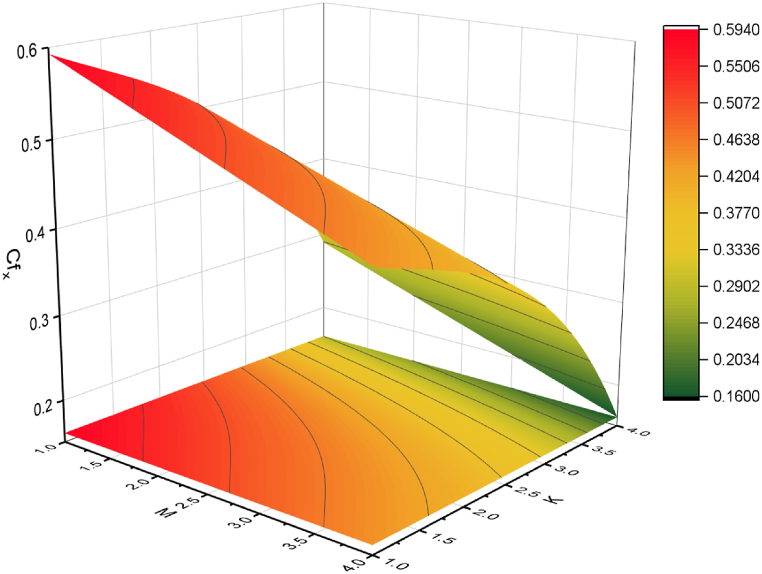
Impact of skin friction on M and K.

**Fig. 12:**
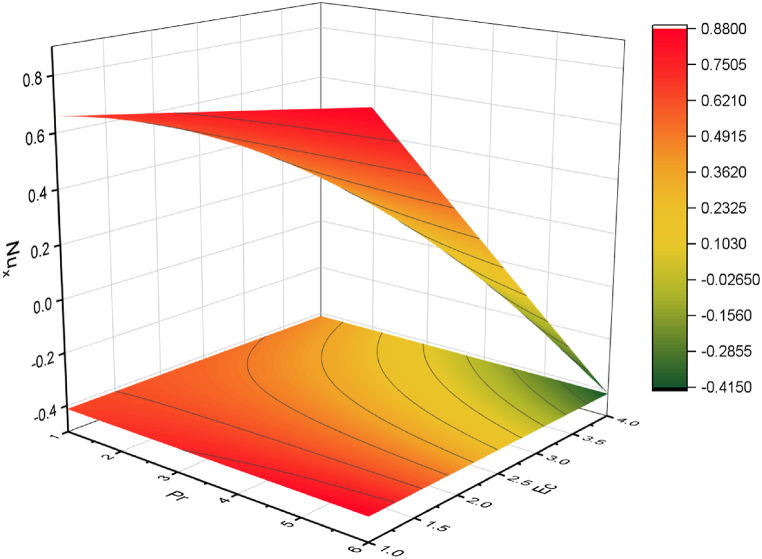
Effect of nusselt number on Pr and Ec.

The objective of this study is to perform a thorough analytical examination of nanofluid flow while accounting for the effects of couple stress. More specifically, the nanofluids under consideration are graphene oxide-ethylene glycol and graphene oxide-water. This analysis is conducted over a mobile surface and takes into account the presence of both a magnetic field and varying viscosity. The effect of various parameters including magnetic field parameter, couple stress parameter, thickness of the liquid, Prandtl number, dynamic viscosity and Eckert number are presented with the help of graphs for both temperature and velocity profiles. The outcomes of this analysis are graphically represented in Fig. 4, Fig. 5, Fig. 6, Fig. 7, Fig. 8, Fig. 9, Fig. 10, Fig. 11, Fig. 12. The influence of distinct factors on the velocity profile is portrayed in Fig. 4, Fig. 5, Fig. 6, Fig. 7, while their impact on temperature distribution is illustrated in Fig. 8, Fig. 9, Fig. 10. Moreover, Fig. 11, Fig. 12 elucidate the consequences of different factors on the Nusselt number and skin friction. For verification, the convergence of the velocity and temperature equations is demonstrated in Table 1, Table 2. In several applications, the GO-EG and GO-W nanofluids in industries and engineering sectors are used to enhance heat transfer ratio. On a stretched surface, research was done while accounting for magnetic and viscous dissipation effects. The Nonlinear Differential Equations (NDEs) in the study were solved utilizing the (HAM), which is a useful analytical technique. The influence of different factors on the skin friction and Nusselt number was examined and showed in Fig. 11, Fig. 12. The analysis in Fig. 11 displays that increasing the values of the pair stress and magnetic field parameters leads to higher skin friction. Fig. 12 presents the impact of the Eckert number and Prandtl number on the Nusselt number. By this we get that as the Eckert and Prandtl numbers increase, the Nusselt number also increases. This indicates that the Eckert number and Prandtl number are positively correlated with the Nusselt number, meaning that higher values of these parameters result in higher Nusselt numbers.

**Table 1:** Illustration of method convergence of $f^{\prime }\left(\eta \right)$.

m	$f^{\prime }\left(\eta \right)$
**5**	$0.0431\times 10^{-1}$
**10**	$0.1014\times 10^{-2}$
**15**	$0.2443\times 10^{-3}$
**20**	$0.0298\times 10^{-5}$
**25**	$0.5783\times 10^{-7}$

**Table 2:** Illustration of method convergence for θ(η).

m	θ(η)
**5**	$0.6391\times 10^{-1}$
**10**	$0.0166\times 10^{-3}$
**15**	$0.9238\times 10^{-5}$
**20**	$0.0626\times 10^{-6}$
**25**	$0.7433\times 10^{-9}$

For both the velocity and temperature distribution, the results are shown in Table 1, Table 2 together with the convergence control value for the particular issue up to the 25th iteration. In Table 3, a comparative analysis is performed between different values of Nusselt number and skin friction. Fig. 4 is presented to observe the influence of the couple stress parameter 0≤K≤0.8, while keeping the remaining parameters constant, on the velocity profiles of both GO−EG, and GO−W fluids. It is evident from Fig. 4 that as the values of *K* increase, the velocity profile diminishes. This reduction in the flow pattern is attributed to the rise in viscous forces associated with the escalating values of K. Thus, the couple stress factor reduces boundary layer thickness, and enhances thermal boundary layer and temperature distribution, this is utilized in industries sector for hotness effect. Fig. 5 is illustrated to noticed the effects of magnetic field factor 0≤M≤0.8, keeping the other factor fixed on velocity profiles of both GO−EG, and GO−W fluids. According to the observations in Fig. 5, shows that velocity profile declines as the values of parameter *M*'s increase. This decrease in the flow field is attributed to the generation of Lorentz forces opposing the flow as the values of M grows. Hence, the presence of the magnetic field factor leads to a decrease in both the boundary layer thickness and velocity, while fostering an expansion in the thermal boundary layer and temperature distribution. Such a flow configuration can find application for heat-related purposes within the industrial sector. The variation in liquid thickness and its influence on the velocity profile can be clearly observed in Fig. 4 for both GO−EG and GO−W on $f^{\prime }\left(\eta \right)$. Furthermore, Fig. 6 clearly depicts that the velocity exhibits a decreasing trend as the liquid thickness increases.

**Table 3:** Compression of Nu , Nusselt number and $C_{f}$, skin friction with the published work.

K	M	β	α	Ec	Pr	$C_{f}$ [42]	$C_{f}$		Nu [42]		Nu	
**0.70**						−1.5618		−1.3416		2.2541		3.0153
**0.75**						−1.8399		−1.4071		1.8082		2.1329
**0.80**						−2.0811		−1.7023		1.5396		1.9531
	5.5					−1.5618		−1.1023		2.2541		2.7519
	6.0					−1.5618		−1.3210		1.5427		1.9036
	6.5					−1.5618		−1.0631		1.1579		1.7628
		0.01				−1.5617		−1.4091		2.3523		2.9541
		0.02				−1.3659		−0.9961		2.5671		3.0081
		0.03				−1.2659		−0.7304		2.7666		3.2671
			0.10			−1.5618		−1.2741		2.2541		2.9615
			0.15			−1.6491		−1.4391		2.0925		2.8612
			0.20			−1.7540		−1.4613		1.9263		2.3091
				3		−1.1235		−0.9971		0.1209		1.5023
				5		−1.5618		−1.1591		2.2541		2.9714
				7		−1.9189		−1.4914		4.2040		4.9871
					1.0	−1.5618		−1.2961		2.2541		2.7541
					1.5	−1.7475		−1.3410		3.2470		3.9841
					2.0	−1.9189		−1.5925		4.2040		4.9361

This condition arises due to the emergence of a resistive force of this nature which strengthens as the liquid thickness increases. This force acts in opposition to the fluid motion and reduces the movement of the fluid within the boundary layer. This flow configuration can be harnessed for heat-related applications in the industrial sector, serving specific important purposes. Fig. 7 is plotted to note the effects of dynamic viscosity 0≤α≤0.8, keeping the other parameters fixed on velocity profiles of both GO−EG, and GO−W fluids. Based on the findings in Fig. 7, it was noticed that the profile of the velocity declines with the increase of the input value α. The decrease in the flow field can be attributed to the presence of viscous forces that oppose the flow, which becomes more prominent with increasing values of α. Consequently, the dynamic viscosity leads to a decrease in both the fluid's velocity and boundary layer thickness. Simultaneously, it encourages a more pronounced distribution of temperature and a broader thermal boundary layer. The impact of the Prandtl number on the temperature distribution for both Case GO−EG, and GO−W are illustrated in Fig. 8. It is clear from the obtained results that Pr on θ(η)0≤Pr≤0.8, keeping the other parameters fixed have an inverse relationship, or the fluid temperature will likewise fall as the Prandtl number decreases. Due to the inverse connection between the thermal diffusivity and Prandtl number, a high Prandtl number denotes a lower thermal diffusivity. Because the ratio of viscous diffusion rate to thermal diffusion rate is large in flow regimes with high Prandtl numbers, viscous diffusion predominates over thermal diffusion, causing a reduction in temperature distribution.

Fig. 9 illustrates how the temperature distribution 0≤Ec≤0.8, is influenced by the Eckert number, while keeping the other parameters fixed for both parameters GO−EG, and GO−W. The provided figure demonstrates a clear link between the Eckert number and the distribution of temperature. It indicates that an increase in the Eckert number leads to a corresponding rise in temperature. This connection is attributed to the Eckert number, which signifies the ratio between kinetic energy and enthalpy. Consequently, an elevated Eckert number signifies greater kinetic energy, resulting in an amplified temperature distribution within the system. Fig. 10 depicts the effect of dynamic viscosity on temperature distribution 0≤α≤0.8, with other parameters held constant for both GO−EG, and GO−W. The figure demonstrates that higher values of dynamic viscosity lead to an increased temperature distribution within the fluids. This can be attributed to the greater kinetic energy of the fluid particles resulting from increased friction forces. Therefore, the temperature of the fluid increases, leading to an enhanced temperature distribution throughout the system.

## 7 Conclusions

The focus of this research was the analytical examination of the flow attributes of nanofluids, with specific emphasis on the graphene oxide-ethylene glycol and graphene oxide-water mixtures. These nanofluids were studied over a mobile surface under the influence of a magnetic field and varying viscosity. By considering the thermophysical characteristics of both the foundational fluid and nanofluid, as well as applying similarity transformations within the governing equations that describe energy and momentum, the study formulated a velocity profile third-order nonlinear ordinary differential equation and a temperature distribution second-order nonlinear ordinary differential equation. An analytical method, known as HAM, was employed to solve these derived equations. Graphical representations were employed to visually elucidate the discussions regarding the solutions obtained through HAM. Convergence verification was carried out through tables and graphs. The main outcomes from the investigation are outlined as follows.

1. The velocity profile exhibits a decrease as the thickness of the liquid increases.
2. A growth in the value of the magnetic field factor causes a decline in the velocity profile.
3. The profile of velocity experiences a decrease with a growth in the value of dynamic viscosity.
4. Increase in the couple stress parameter, causes decline in the velocity profile.
5. The temperature profile experiences a decline with a growth in the value of the Prandtl number.
6. An elevation in the Eckert number results in an enhancement of the temperature distribution.
7. The temperature profile is amplified as the dynamic viscosity value increases.
8. Increasing the magnetic field and couple stress factor leads to an augmentation in the skin friction.
9. Elevating the Prandtl number and Eckert number values leads to an enhancement in the Nusselt number.

## Data availability statement

No data was used for the research described in the article.

## CRediT authorship contribution statement

**Ali Rehman:** Writing – original draft, Visualization, Software, Resources, Methodology, Investigation, Formal analysis, Data curation, Conceptualization. **Ma Chau Khun:** Writing – review & editing, Validation, Supervision, Project administration, Methodology, Investigation, Conceptualization. **Zabidin Salleh:** Writing – review & editing, Validation, Supervision, Resources, Project administration, Investigation, Funding acquisition. **Waris Khan:** Visualization, Validation, Software, Methodology, Investigation, Formal analysis, Data curation, Conceptualization. **Maryam Sulaiman Albely:** Visualization, Software, Resources, Methodology, Investigation, Formal analysis. **Rashid Jan:** Visualization, Validation, Methodology, Investigation, Formal analysis, Data curation, Conceptualization. **Somayah Abdualziz Alhabeeb:** Writing – review & editing, Visualization, Validation, Software, Resources, Methodology, Investigation, Formal analysis, Data curation.

## Declaration of competing interest

The authors declare that they have no known competing financial interests or personal relationships that could have appeared to influence the work reported in this paper.

## Nomenclature

**Symbol Description**
$u_{w}$: Stretching**/**Shrinking velocity $\left(ms^{-1}\right)$
uandv: x and y components of velocity $\left(ms^{-1}\right)$
K: Couple stress parameter
T: Temperature of hybrid nanofluid (K)
b: Rate constant for stretching
$T_{\infty }$: Ambient temperature (K)
Ec: Eckert number
$T_{w}$: Wall temperature (K)
α: Dynamic viscosity
$Re_{x}$: Reynold number
$\rho_{hnf}$: Hybrid nanofluid density $\left(kgm^{-3}\right)$
w: Surface condition
σ: Electrical conductivity of the base fluid $sm^{-1}$
${\left(\rho C_{p}\right)}_{nf}$: Specific heat of Nano-fluid $\left(m^{2}s^{-2}K^{-1}\right)$
$\lambda =\frac{u_{w}}{U_{w}}$: fused velocity
$\nu_{f}$: Kinematic viscosity $\left(m^{2}s^{-1}\right)$
η: Similarity variable
$k_{nf}$: Thermal condictivity of nanofluid $\left(m^{2}s^{-1}\right)$
φ: Volume fraction of nanoparticles
nf: Nanofluid
f: Base fluid
PDE's: Partial differential equations
OHAM: Optimal Homotopy analysis method
GO−EG: Graphene Oxide Ethylene Glycol

**Symbol Description**
M: Magnetic factor
x,y: Plane coordinate axis
$C_{fx}$: Coefficient of skin friction
$\tau_{w}$: Shear stress
$B_{0}$: Magnetic field strength $\left(Am^{-1}\right)$
$Nu_{x}$: Nusselt number
Q: Heat source/sink factor
$E_{0}$: Electric field strength $\left(Vm^{-1}\right)$
E: Parameter of electric field (N/C)
$f^{\prime }$: Velocity without dimension
∞: Ambient condition
$\nu_{nf}$: Kinematic viscosity of nanofluid $\left(m^{2}s^{-1}\right)$
$\mu_{nf}$: Viscosity of nanofluid $\left(kgm^{-1}s^{-1}\right)$
Pr: Prandtl number
$\rho_{nf}$: Density of nanofluid $\left(kgm^{-3}\right)$
nf: nanofluid
GO-W: Graphene Oxide - Water
ODE's: Ordinary differential equations
MHD: Magnetohydrodynamic
(NODE): Nonlinear Ordinary Differential Equation
